# Draft Genome Sequences of Three Salmonella enterica Serovar 4,[5],12:i:− Strains and One S. enterica Serovar Typhimurium Strain, Isolated in Brazil

**DOI:** 10.1128/genomeA.00488-18

**Published:** 2018-07-05

**Authors:** Ana Isabela Lopes Sales, Guilherme Paier Milanez, Leandro C. Nascimento, Camila Pinheiro do Carmo, Fernanda Luz Paulino da Costa, Gonçalo Amarante Guimarães Pereira, Roberto Martinez, Marcelo Brocchi

**Affiliations:** a Departamento de Biologia Celular, Molecular e Bioagentes Patogênicos, Faculdade de Medicina de Ribeirão Preto, Universidade de São Paulo (USP), Ribeirão Preto, SP, Brazil; b Departamento de Genética, Evolução Microbiologia e Imunologia, Instituto de Biologia, Universidade Estadual de Campinas (UNICAMP), Campinas, SP, Brazil; c Laboratório de Tecnologias de Alto Desempenho (LaCTAD), Universidade Estadual de Campinas (UNICAMP), Campinas, SP, Brazil; d Departamento de Clínica Médica, Faculdade de Medicina de Ribeirão Preto, Universidade de São Paulo (USP), Ribeirão Preto, SP, Brazil

## Abstract

Draft genomes of three Salmonella enterica 4,[5],12:i:− (STi) strains isolated from human infections were obtained using Illumina sequencing. They were negative for the *fljBA* operon but positive for *hin*, and k-mer analyses revealed their identity as S. enterica 4,[5],12:i:− 08-1736 and *S*. Typhimurium. A draft *S*. Typhimurium sequence is described for comparison.

## Abstract (precis)

Draft genomes of three Salmonella enterica 4,[5],12:i:− (STi) strains isolated from human infections were obtained using Illumina sequencing. They were negative for the *fljBA* operon but positive for *hin*, and k-mer analyses revealed their identity as S. enterica 4,[5],12:i:− 08-1736 and *S*.

## GENOME ANNOUNCEMENT

Salmonella enterica, an important human and animal pathogen (1, 2), is often associated with antibiotic resistance (3). This species is classified into different serotypes according to its somatic, flagellar, and, when expressed, capsular antigens (4). It is classified as either typhoid Salmonella (TS) or nontyphoid Salmonella (NTS) based on the serovar and host specificity. S. enterica subsp. enterica serovar Typhimurium (antigenic formula S. enterica I 4,[5],12:i:1,2) is among the most frequently identified NTS serovars isolated from human infections (2). Characteristics of this serovar include the nonconcomitant expression of two flagellar antigens (flagellar phases I and II), encoded by the *fliC* and *fljB* genes, respectively (5). Expression of *fliC* is negatively regulated by the *fljA* gene product, which forms an operon with *fljB* (6–8). The promoter of the *fljBA* operon extends into the *hin* sequence, a DNA fragment that is able to invert its orientation. Therefore, the type of flagellar antigen expressed is governed by the orientation of *hin* (5). Since the last century, *S*. Typhimurium variants unable to express flagellar phase II antigen have been isolated at an increased rate, becoming emergent an S. enterica serotype (9). S. enterica 4,[5],12:i:− originated from *S*. Typhimurium through the deletion of the *fljBA* operon. Different clones are distinguished based on the molecular events responsible for the *fljBA* deletion (9). Here, we describe the draft genome of three strains of S. enterica 4,[5],12:i:− (607STi, 633STi, and 691STi) and one of *S*. Typhimurium (662ST), all isolated in Brazil from human infections. Genomic DNA was prepared using the Wizard R genomic DNA purification kit (Promega, USA) according to manufacturer’s instructions. Genomic DNA integrity was assessed by electrophoresis on 1% agarose gel. Library preparation was performed using the Nextera paired-end sample preparation kit and TruSeq DNA PCR-free LT sample prep kit and processed in a HiSeq 2500 system (Illumina, San Diego, CA). Genome assembly was performed with Velvet (10) and annotation with RAST (11).

The number of contigs was 30 or 36, and the mean scaffold length was 134,767 to 163,767 bp. The GC content of all strains was 52.2%. Bacterial typing was performed with MLST 1.7, a platform for multilocus sequence typing (MLST) from assembled genomes (12). All strains were typed as sequence type 19 (ST-19), although the *S*. Typhimurium strain did not match perfectly for the *thra* locus due to a 1-base substitution. The ST-19 type is common for *S*. Typhimurium.

The prediction of prokaryotic species by k-mer genomic comparisons using KmerFinder 2.0 (13) indicated high scores for S. enterica 4,[5],12:i:− strain 08-1736, a 4,[5],12:i:− strain, and other *S*. Typhimurium strains. Scores for other S. enterica serovars were lower. BLAST analyses indicated an absence of the *fljBA* operon in the S. enterica 4,[5],12:i:− strains, though the *hin* sequence was present. The *S*. Typhimurium strain was positive for both sequences.

In conclusion, the three sequenced 4,[5],12:i:− strains presented high identity to S. enterica 4,[5],12:i:− strain 08-1736 and *S*. Typhimurium. Further analyses are under way to characterize this *S*. Typhimurium variant serovar in Brazil.

### Accession number(s).

This whole-genome shotgun project has been deposited in GenBank under the accession no. QAWG00000000, QAWH00000000, QAWI00000000, and QAWJ00000000. The versions described in this paper are the first versions, QAWG01000000, QAWH01000000, QAWI01000000, and QAWJ01000000.

## References

[1] Andrews-Polymenis HL, Bäumler AJ, McCormick BA, Fang FC 2010 Taming the elephant: *Salmonella* biology, pathogenesis, and prevention. Infect Immun 78:2356–2369. doi:10.1128/IAI.00096-10. 20385760 PMC2876576

[2] Crump JA, Sjölund-Karlsson M, Gordon MA, Parry CM 2015 Epidemiology, clinical presentation, laboratory diagnosis, antimicrobial resistance, and antimicrobial management of invasive *Salmonella* infections. Clin Microbiol Rev 28:901–937. doi:10.1128/CMR.00002-15. 26180063 PMC4503790

[3] Fernandes SA, Camargo CH, Francisco GR, Bueno MFC, Garcia DO, Doi Y, Casas MRT 2017 Prevalence of extended-spectrum β-lactamases CTX-M-8 and CTX-M-2-producing *Salmonella* serotypes from clinical and nonhuman isolates in Brazil. Microb Drug Resist 23:580–589. doi:10.1089/mdr.2016.0085. 27828759

[4] Agbaje M, Begum RH, Oyekunle MA, Ojo OE, Adenubi OT 2011 Evolution of *Salmonella* nomenclature: a critical note. Folia Microbiol 56:497–503. doi:10.1007/s12223-011-0075-4. 22052214

[5] McQuiston JR, Fields PI, Tauxe RV, Logsdon JM Jr 2008 Do *Salmonella* carry spare tyres? Trends Microbiol 16:142–148. doi:10.1016/j.tim.2008.01.009. 18375124

[6] Bonifield HR, Hughes KT 2003 Flagellar phase variation in *Salmonella enterica* is mediated by a posttranscriptional control mechanism. J Bacteriol 185:3567–3574. doi:10.1128/JB.185.12.3567-3574.2003. 12775694 PMC156219

[7] Aldridge P, Gnerer J, Karlinsey JE, Hughes KT 2006 Transcriptional and translational control of the *Salmonella fliC* gene. J Bacteriol 188:4487–4496. doi:10.1128/JB.00094-06. 16740955 PMC1482933

[8] Yamamoto S, Kutsukake K 2006 FljA-mediated posttranscriptional control of phase 1 flagellin expression in flagellar phase variation of *Salmonella enterica* serovar Typhimurium. J Bacteriol 188:958–967. doi:10.1128/JB.188.3.958-967.2006. 16428400 PMC1347349

[9] Switt AIM, Soyer Y, Warnick LD, Wiedmann M 2009 Emergence, distribution, and molecular and phenotypic characteristics of *Salmonella enterica* serotype 4,5,12:i:−. Foodborne Pathog Dis 6:407–415. doi:10.1089/fpd.2008.0213. 19292687 PMC3186709

[10] Zerbino DR, Birney E 2008 Velvet: algorithms for de novo short read assembly using de Bruijn graphs. Genome Res 18:821–829. doi:10.1101/gr.074492.107. 18349386 PMC2336801

[11] Overbeek R, Olson R, Pusch GD, Olsen GJ, Davis JJ, Disz T, Edwards RA, Gerdes S, Parrello B, Shukla M, Vonstein V, Wattam AR, Xia F, Stevens R 2014 The SEED and the Rapid Annotation of microbial genomes using Subsystems Technology (RAST). Nucleic Acids Res 42:D206–D214. doi:10.1093/nar/gkt1226. 24293654 PMC3965101

[12] Larsen MV, Cosentino S, Rasmussen S, Friis C, Hasman H, Marvig RL, Jelsbak L, Sicheritz-Pontén T, Ussery DW, Aarestrup FM, Lund O 2012 Multilocus sequence typing of total-genome-sequenced bacteria. J Clin Microbiol 50:1355–1361. doi:10.1128/JCM.06094-11. 22238442 PMC3318499

[13] Larsen MV, Cosentino S, Lukjancenko O, Saputra D, Rasmussen S, Hasman H, Sicheritz-Pontén T, Aarestrup FM, Ussery DW, Lund O 2014 Benchmarking of methods for genomic taxonomy. J Clin Microbiol 52:1529–1539. doi:10.1128/JCM.02981-13. 24574292 PMC3993634

